# Supplementary material for: Distribution of Hepatitis B prevention services in Wakiso District, Central Uganda
Source: PLOS Glob Public Health. 2023 Sep 22;3(9):e0000478. doi: 10.1371/journal.pgph.0000478 (PMC10516414; doi:10.1371/journal.pgph.0000478)
Supplement: S1 Text — (PDF) [file pgph.0000478.s001.pdf]

Hepatitis B service availability and readiness Assessment in Wakiso district

Name of Health Facility

____

Level of health facility

- Hospital
- Health Centre IV
- Health Centre III

Ownership of health facility

- Public
- Private
- Private Not For Profit (PNFP)
- Private for Profit (PFP)

Location of the health facility

- Rural
- Urban

GPS coordinates

____

latitude (x.y °)

longitude (x.y °)

altitude (m)

accuracy (m)

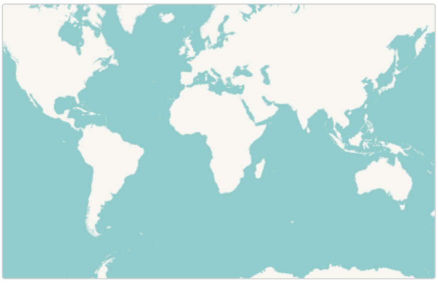

Name of Health sub district

- Busiro North
- Busiro East
- Busiro south
- Kyadondo North
- Kyadondo south
- Kyadondo East
- Entebbe municipality

Did this health facility receive any hepatitis B vaccine doses in the last 12 months? (Check delivery records for proof)

- Yes
- No

Does the health facility have refrigerators for keeping vaccines?

- Yes
- No

Do you have a cold chain technician/ assistant at this health facility?

- Yes
- No

Has any of the cold chain technicians/ assistants been trained in temperature monitoring for hepatitis B vaccines?

- Yes
- No

Is the hepatitis B vaccine available in this facility?

- Yes
- No

Are there guidelines to follow when exposed to hepatitis B in your facility?

- Yes
- No

Are there testing kits for hepatitis b in your facility?

- Yes
- No

Are HB testing kits in stock (check in the store)?

- Yes
- No

Does the health facility have a routine vaccination schedule for the health care providers?

- Yes
- No

Does the health facility have any infection control promotion materials?

- Yes
- No

Are there reminders and/or job aids posted that promote safe use of injections at this facility?

- Yes
- No

Has this health facility held any CMEs on hepatitis B in the last 12 months? (Look out for evidence e.g. CME schedule or)

- Yes
- No

Does the health facility have colour coded waste bins? (Observe)

- Yes
- No

Is there any infectious waste other than used sharps (for example, bloody swabs or dressings) that is not in an appropriate container? (observe)

- Yes
- No

Does the health facility have any staff trained in hepatitis B testing?

- Yes
- No

Has this health facility had someone trained in management of hepatitis B in the last 12 months?

- Yes
- No

Does the health facility have an infection control focal person?

- Yes
- No

Does the health facility have hand washing facilities? (Observe)

- Yes
- No

Do the HWFs have soap?

- Yes
- No

Does the health facility have PEP guidelines in case of injuries?

- Yes
- No
