# Supplementary material for: Distribution of Hepatitis B prevention services in Wakiso District, Central Uganda
Source: PLOS Glob Public Health. 2023 Sep 22;3(9):e0000478. doi: 10.1371/journal.pgph.0000478 (PMC10516414; doi:10.1371/journal.pgph.0000478)
Supplement: Attachment — Submitted filename: Response to Reviewers.docx [file pgph.0000478.s003.docx]

**Response to comments**

**Reviewer #1:**  This is a cross-sectional descriptive study conducted in 55 healthcare facilities to assess service availability of hepatitis B vaccine and testing services in Wakiso District, Central Uganda. Based on the assessment the hepatitis B vaccine was available in only 27.3 %, while testing services were available 60% the facilities. Based on descriptive analysis, level of health care facility and location showed significant association with availability of hepatitis B vaccine. While hepatitis B testing services was associated with level of healthcare facility, ownership and location. This is an important area to be studied and the results of this study inform the Wakiso District. Nonetheless the number of districts included the study is very small, the analysis used is not in congruent with the title, even so the authors only did a univariate analysis with our controlling the potential confound effect of the variables one another.

**Response:** Thank you. Despite being a single study site, Wakiso was selected because of the following reasons; 1) It is the most populated district in Uganda, 2) It surrounds Kampala city, Uganda’s capital, and hosts a bulk of the urban population. Besides, it also has rural settlements which provides an opportunity for comparison. In addition, Wakiso district has the highest number of healthcare facilities in Uganda, making it an ideal site for the study. Also, the fact that Wakiso has both an urban and rural setting which provided an opportunity for comparison. Regarding the title, we have changed it to reflect the analysis used. We have also made changes throughout the manuscript.

**Specific comments
Comment**: The title ‘Geospatial distribution of Hepatitis B prevention services in Wakiso District, Central Uganda’, is misleading as such there is no geospatial analysis conducted in this study. The only thing here is using the coordinates the location of the health facilities has been mapped. Better to remove the term ‘geospatial’ or conduct appropriate rigorous analysis.

**Response:** Thank you. The title of the study has been changed to ‘Distribution of Hepatitis B prevention services in Wakiso District, Central Uganda’

**Comment**: What is the key message of this paper ‘Healthcare facilities offering vaccination and testing services were mostly in urban healthcare facilities’, is that not clear that facilities in urban area are better off in-service provision overall, not particular to this analysis?

**Response:** Thank you. We have edited this. The key message is hepatitis B prevention services were sub-optimal across all healthcare facility levels, locations, and ownership.

**Comment**: The assertion ‘close to Kampala, Uganda’s capital’ is not supported by the available data. Were there measurements in terms of distance of the health facilities from Kampala? It seems most of the health facilities included in the study are close to Kampala.

**Response**: A scale has been provided in the revised maps to show the proximity of the facilities to Kampala. A heat map has also been provided to illustrate the proximity of healthcare facilities to Kampala.`

**Comment**: There is no clear justification of selection of 55 (9%) health facilities out of the 589 HCFs in the district. Does this give enough sample size to make the conclusion the authors and making in the manuscript? The allocation of the sample does not look proportional for example, although 477(81%) health facilities were owned privately only few were sampled (47%).

**Response**: The study selected only healthcare facilities that provided high-risk medical interventions such as blood transfusions, delivering higher numbers of mothers, and other surgical procedures that can elevate the risk of transmission of HBV (Lines 172-175). The 477 private facilities also included clinics and level II facilities which don’t offer high-risk medical interventions (and thus were excluded). Thus, we selected only 26 because these met the inclusion criteria of providing high-risk medical interventions.

**Comment**: The number of hospitals included in the study differs between the text (line 172, indicates 6) and the table 1 (indicates 4).

**Response**: Thank you. This has been rectified (Line 172)

**Comment**: The variables measured are very limited. The authors should have developed a conceptual framework with clear indication of proximal and distal factors which affect the service availability.

**Response**: We have acknowledged this as one of the limitations of the study. (Lines 363)

**Comment**: The analysis done is univariate analysis, it is worth doing multiple logistic regression analysis to assess factors independently associated with the services.
**Response**: We didn’t conduct multivariate analysis because the assumptions were not met. There was also multicollinearity between the variables; the independent variables were highly correlated with each other, which made it inappropriate to conduct multiple logistic regression analysis. Besides, our objective was to illustrate the distribution of hepatitis B services across the different healthcare facility characteristics.

**Comment**: Table 2 needs rework, the outcome of the analysis should be availability of the hepatitis B vaccine and testing services. Some of the variables include could be explanatory variables such as availability of refrigerator, presence of trained man power etc.

**Response**: Table 2 has been modified to indicate service readiness and availability of hepatitis B. Our intention was to show how a set of readiness and service availability indicators varied across healthcare facility levels, ownership and location.

**Reviewer #2**

**Comment:** Is it that the distribution of care and treatment services for hepatitis B prevention only unknown in Wakiso district in the whole country as alluded? What makes Wakiso district a unique focus in the entire country relative to all the other districts in this study?
**Response**: Wakiso was selected because of the following reasons; 1) It is the most populated district in Uganda, 2) It surrounds Kampala city, Uganda’s capital, and hosts a bulk of the urban population and rural settlements in the periphery. In addition, Wakiso district has the highest number of healthcare facilities in Uganda, making an ideal site for the study. Also, the fact that Wakiso has both an urban and rural setting provided an opportunity for comparison.

**Comment**: Under study setting, a map of Wakiso district with its main features and its position relative to Uganda would be useful in simplifying the details in this section and orienting the reader who is not familiar with Uganda.

**Response**: A map of Uganda showing location of Wakiso district has been added.

**Comment**: As the questionnaire were informed to some extents by SARA, the authors should comment whether there is a SARA or availability of other surveys that have collected similar information for hepatitis B in Uganda.
**Response**: Thank you. To the best of our knowledge, no similar surveys have been conducted in Uganda. This strength has been acknowledged in the strengths and limitations section.

**Comment**: Provide an improved description of the checks that were done to verify the quality of the GPS data. At the moment only a summary is given and does not detail the actual checks that we conducted such as accuracy of the location, GPS coordinates being next to a structure etc.
**Response**: A description of the quality control and assurance checks has been added. Briefly, the research assistants picked GPS coordinates from each point which ensured accuracy. Data were visually inspected to detect any errors. The locations were mapped as Keyhole Markup Language (KML) and visualized on a base map in google earth to ensure visual accuracy. (Lines 217-221)

**Comment**: The section on Geospatial distribution requires more work. Creating a shapefile (vector file) from the collected GPS data is not necessary to describe as it’s a routine process. However, what could be useful to describe if any geoprocessing was done to the collected variables beyond visualizing them on map so that the section can stand on its own. At the moment its only describing how the GPS data was collected and creation of shapefile which can be summarized in 2-3 lines. If the sampling was representative and random, clustering analysis, interpolation among other geospatial techniques would have been useful in providing subnational metrics in this district.
**Response**: Thank you. The description of the creation of a shapefile has been omitted. We performed interpolation using Kernel density estimation to produce a heatmap showing clustering of healthcare facilities. High clustering was observed in close proximity to Kampala city (Line 231).

**Comment**: My second major concern is to what extent can the results of purposively selected facilities be deemed to show the geospatial distribution of outcomes and proportions in a district? Would these maps and statistics not be misleading to the audience? The results are even further stratified by rural/urban, private/public facilities from a purposive survey. Since the survey is random would these proportions and maps not be biased?
**Response**: The purposive sampling was employed in the selection of facilities that provided high-risk medical interventions such as blood transfusions, delivering higher numbers of mothers, and other surgical procedures that can elevate the risk of transmission of HBV.

**Comment**: I would suggestion removing details about the geospatial patterns in the manuscript and in the title since no clustering analysis was done. This is because only the location of the health facilities was collected and visualized in map together with the associated attributes from non-random survey.

**Response**: Thank you. The title of the study has been changed to ‘Distribution of Hepatitis B prevention services in Wakiso District, Central Uganda.’ This has also been done throughout the paper.
