# Supplementary material for: Distribution of Hepatitis B prevention services in Wakiso District, Central Uganda
Source: PLOS Glob Public Health. 2023 Sep 22;3(9):e0000478. doi: 10.1371/journal.pgph.0000478 (PMC10516414; doi:10.1371/journal.pgph.0000478)
Supplement: Attachment — Submitted filename: Response to comments_Distribution of HBV services.docx [file pgph.0000478.s004.docx]

**Review Comments to the Author
Please use the space provided to explain your answers to the questions above. You may also include additional comments for the author, including concerns about dual publication, research ethics, or publication ethics. (Please upload your review as an attachment if it exceeds 20,000 characters)**

**Thank you, reviewers, for the feedback that has definitely made this paper better. Below is our response to the comments raised during the review.**

Reviewer #2: "We did not conduct multivariate analysis to control for confounding because the data did not fulfil the required assumptions i.e., multicollinearity, and independence of observations (29, 30)." This does not justify why multivariate analysis was not done. However, if the intention was to illustrate the availability and distribution of HBV prevention services across the different healthcare facility characteristics then the details on multivariate should be omitted.

**Response:** Thank you for the feedback. Indeed, our intention was to illustrate the availability and distribution of HBV prevention services across the different healthcare facility characteristics. Page 10 Lines 191-194

**Comment:** One of my previous comments was only addressed partly: "Comment: My second major concern is to what extent can the results of purposively selected facilities be deemed to show the distribution of outcomes and proportions in a district?
Would these maps and statistics not be misleading to the audience? The results are even further stratified by rural/urban, and private/public facilities from a purposive survey. Since the survey is random would these proportions and maps not be biased?

**Response:** Thank you. The purposive selection was only done in the selection of healthcare facilities that, by their level and nature of activities were expected to offer vaccination services. For example, hospitals, healthcare facilities (levels III and IV), and private healthcare facilities that offer delivery services (and otherwise at a similar level as health centre IIIs or IVs). Thus, smaller healthcare facilities such as drug shops, which by their level of service provision, are not expected to offer vaccination, were excluded. Ideally, we surveyed all healthcare facilities, which by their level were expected to offer vaccination services.

**Reviewer #3**: The article seems pertinent to the objectives that were set to determine. It would be convenient if they could specify the on-site verification of the information obtained through the web questionnaire. Also, in having more information on the availability of vaccines, human resources, and determinants for the lack of access to vaccination, which is the most cost-effective strategy to avoid chronic HBV infection and its sequelae such as cancer and cirrhosis.

Response: Thank you. Interviews were done on-site and thus verification was performed during the course of data collection. Internet was only needed to upload the data collection tools at the end of the day.
